# A Clustering Approach to Classify Italian Regions and Provinces Based on Prevalence and Trend of SARS-CoV-2 Cases

**DOI:** 10.3390/ijerph17155286

**Published:** 2020-07-22

**Authors:** Andrea Maugeri, Martina Barchitta, Antonella Agodi

**Affiliations:** 1 Department of Medical and Surgical Sciences and Advanced Technologies “GF Ingrassia”, University of Catania, 95123 Catania, Italy; andrea.maugeri@unict.it (A.M.); martina.barchitta@unict.it (M.B.); 2 Azienda Ospedaliero-Universitaria “Policlinico-Vittorio Emanuele”, 95123 Catania, Italy

**Keywords:** COVID-19, clustering, positive cases, epidemiology

## Abstract

While several efforts have been made to control the epidemic of severe acute respiratory syndrome coronavirus 2 (SARS-CoV-2) in Italy, differences between and within regions have made it difficult to plan the phase two management after the national lockdown. Here, we propose a simple and immediate clustering approach to categorize Italian regions working on the prevalence and trend of SARS-CoV-2 positive cases prior to the start of phase two on 4 May 2020. Applying both hierarchical and k-means clustering, we identified three regional groups: regions in cluster 1 exhibited higher prevalence and the highest trend of SARS-CoV-2 positive cases; those classified into cluster 2 constituted an intermediate group; those in cluster 3 were regions with a lower prevalence and the lowest trend of SARS-CoV-2 positive cases. At the provincial level, we used a similar approach but working on the prevalence and trend of the total SARS-CoV-2 cases. Notably, provinces in cluster 1 exhibited the highest prevalence and trend of SARS-CoV-2 cases. Provinces in clusters 2 and 3, instead, showed a median prevalence of approximately 11 cases per 10,000 residents. However, provinces in cluster 3 were those with the lowest trend of cases. K-means clustering yielded to an alternative cluster solution in terms of the prevalence and trend of SARS-CoV-2 cases. Our study described a simple and immediate approach to monitor the SARS-CoV-2 epidemic at the regional and provincial level. These findings, at present, offered a snapshot of the epidemic, which could be helpful to outline the hierarchy of needs at the subnational level. However, the integration of our approach with further indicators and characteristics could improve our findings, also allowing the application to different contexts and with additional aims.

## 1. Introduction

The novel coronavirus (SARS-CoV-2) epidemic began to spread in the Hubei province (China) at the end of 2019, and then to more than 200 countries worldwide [1]. At the end of February 2020, two distinct outbreaks of SARS-CoV-2 occurred in two small Italian areas within the Lombardy and Veneto regions [2]. The epidemic, since then, spread at different times and with different intensities across all the Italian regions [3]. On 10 March 2020, the Italian government has promptly reacted to the epidemic by adopting a first set of national restrictions and recommendations (e.g., travel restrictions, quarantine and contact precautions) [4,5], which were strengthened on 23 March by avoiding non-essential industrial productions and social interactions [4,5]. While these efforts have been made to control the epidemic [4,5], differences between and within regions have made it difficult to plan the phase two management after the national lockdown. As a consequence of the control measures adopted, there has been a 5.3% decrease in SARS-CoV-2 positive patients during the week preceding the beginning of phase two in Italy, 4 May 2020 [3]. This resulted in a total of 100,179 active cases throughout the Italian territory on 3 May 2020 [3]. However, these patients were not evenly distributed across Italian regions, with three regions (i.e., Lombardy, Piedmont, and Emilia-Romagna) that presented more than 60% of the total active cases [3]. Several modelling studies evaluated the efficacy of control measures adopted in Italy and predicted future scenarios to help policymakers in designing the phase two strategy against the SARS-CoV-2 epidemic [6,7,8,9,10]. However, identifying different regional and provincial clusters would be just as crucial to plan strategies for relaxing the restrictions in accordance with specific situations and needs. As for other emerging diseases, useful and innovative tools could be able to provide a snapshot of the epidemic at the regional and provincial level. Here, we used two of the most common clustering methods (i.e., hierarchical clustering and k-means algorithm) [11] to categorize Italian regions and provinces into several groups. At the regional level, this clustering approach works directly on the prevalence and trend of active cases in each region, prior to the start of phase two on 4 May 2020. Specifically, it has been applied to regional data on the prevalence of SARS-CoV-2 positive cases on 3 May 2020 and their trend in the previous week from the 27 April to the 3 May 2020. At the provincial level, we applied a similar approach, but working on the prevalence and trend of total SARS-CoV-2 cases, due to the limited availability of data on positive cases. Thus, our aim was to apply a simple clustering approach in order to classify Italian regions and provinces based on the prevalence and trend of SARS-CoV-2 cases prior to the start of phase two on 4 May 2020.

## 2. Materials and Methods 

We first obtained data on SARS-CoV-2 positive cases and on the number of tests performed, which were collected and released by Italy’s Civil Protection of the Italian Ministry of Health from 27 April to 3 May 2020. These data, deposited on GitHub [12,13], were freely available and provided at the regional and provincial level. On the dedicated website [14], it is also possible to consult the daily prevalence of SARS-CoV-2 cases, their weekly trend and other information on the epidemic in Italy. We also obtained the number of residents of each region and province from the Italian National Institute of Statistics (ISTAT, Istituto Nazionale di Statistica) [15]. Thus, our study did not contain data at the individual level that would require ethical approval. Using the aforementioned data, we calculated the following regional and provincial indicators:

- The regional prevalence of SARS-CoV-2 positive cases on 3 May 2020 (i.e., expressed as the number of positive cases per 10,000 residents);
- The weekly regional trend of SARS-CoV-2 positive cases from 27 April to 3 May 2020 (expressed as the percentage of increment/decrement of positive cases);
- The provincial prevalence of SARS-CoV-2 cases on 3 May 2020 (i.e., expressed as the number of total cases per 10,000 residents);
- The weekly provincial trend of SARS-CoV-2 cases from 27 April to 3 May 2020 (expressed as percentage of increment of total cases);
- The number of tests performed per 10,000 residents (i.e., only at the regional level).

We then inspected the structure of the regional dataset by a three-dimensional scatter plot and tested for normality using the Kolmogorov–Smirnov test. According to the skewed distribution of the aforementioned indicators, we tested for correlations using the Spearman’s rank correlation coefficient. We separately tested the linear relationship of the number of tests performed per 10,000 residents (independent variable) with a prevalence or trend of SARS-CoV-2 cases (dependent variables) using linear regression models. Variables included in the regression models were log-transformed before being analyzed and the results were reported as β and its standard error (SE). Since data on the prevalence and trend of SARS-CoV-2 positive cases were related to different testing strategies between Italian regions, we then used the residuals of linear regressions to adjust both indicators for the number of tests performed per 10,000 residents. To account for different scales between the indicators, the adjusted values were further standardized using the Z-Score formula: z = (x − μ)/σ, where x was the log-transformed indicator, μ was its national log-transformed mean, and σ was the standard deviation. Working on these values (i.e., the log-transformed prevalence and trend of SARS-CoV-2 positive cases), we then applied hierarchical clustering using Ward’s criterion. The distance between regions—and hence their similarity—was determined by calculating their squared Euclidean distance in the two-dimensional plane. We also applied the Euclidean distance but obtaining the same cluster solution (data not shown). This clustering was shown by a hierarchical tree—also called dendrogram—in which the height of the branches indicated the distance between the clusters [11]. The optimal number of clusters—k—was determined using the silhouette method. Specifically, we derived four clustering solutions, which differed for the number of clusters *k,* into a range from two to five clusters (2 ≤ *k* ≤ 5). For each clustering solution, we calculated the silhouette value that represented how each region was cohered to its own cluster and separated from the others. We chose the optimal clustering solution that maximized the silhouette value [16]. Since slight differences in the clustering solution could be obtained using different methods [11], we consolidated the hierarchical clustering by applying the k-means algorithm. This method partitioned *n* observations into *k* clusters by minimizing intra-cluster variation [11]. In contrast to hierarchical clustering, the k-means clustering required a predefined number of clusters, which was determined according to the silhouette method applied to hierarchical clustering. Finally, we evaluated the inter-cluster variability using the Kruskal–Wallis test.

The provincial dataset on the prevalence and trend of total SARS-CoV-2 cases was analyzed in the same way, except for the preliminary adjustment for the number of tests performed. In fact, there were no accurate and complete data on the number of tests performed at provincial level. In brief, the prevalence and trend of total SARS-CoV-2 cases at the provincial level were first log-transformed and standardized using the Z-Score formula. Then, we used hierarchical clustering based on the squared Euclidean distance and Ward’s criterion to derive four clustering solutions (2 ≤ *k* ≤ 5). The optimal clustering solution was chosen to maximize the silhouette value [16]. Finally, we consolidated the hierarchical clustering solution by applying the k-means algorithm with a predefined number of clusters. All the analyses were performed on the SPSS software (version 23.0, SPSS, Chicago, IL, USA), with a significance level α of 0.05.

## 3. Results

### 3.1. Description of Data

The prevalence of SARS-CoV-2 positive cases on 3 May 2020, their trend in the week before, and the number of tests performed in each region are reported in Table 1.

**Figure 1:**
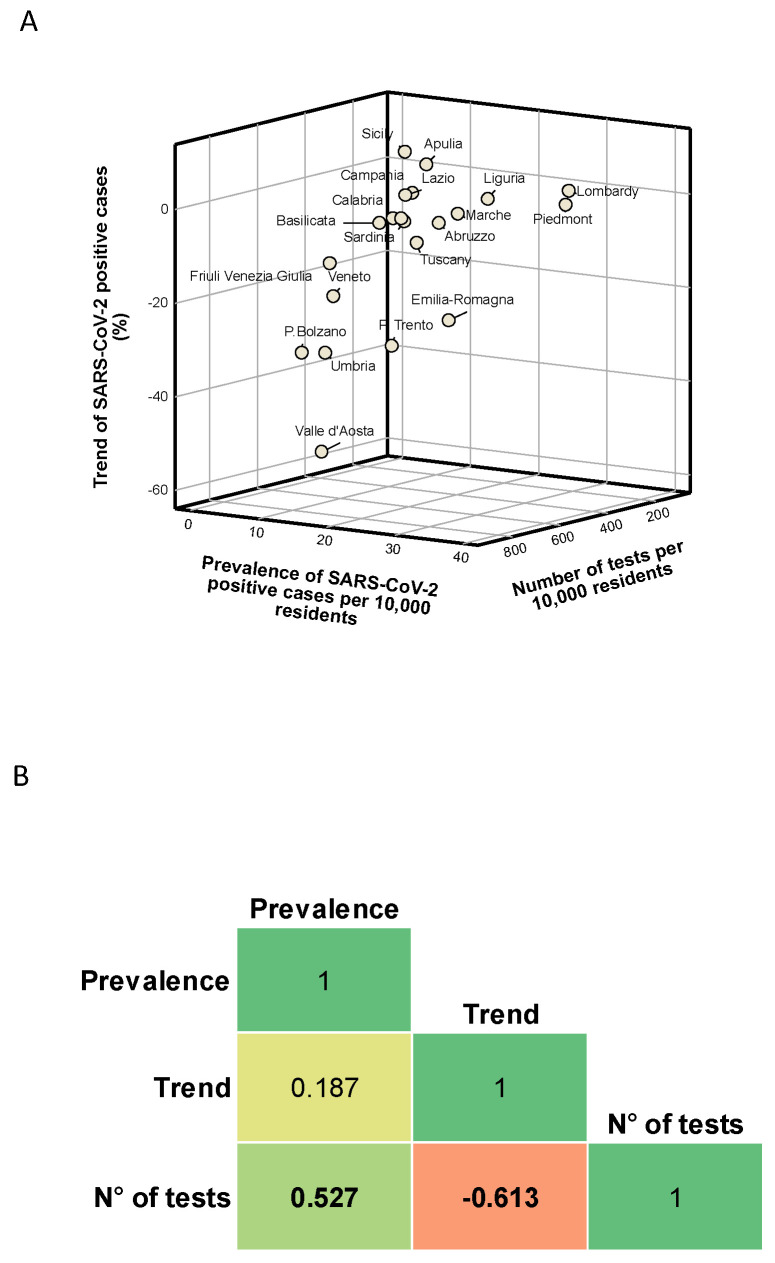
Description and correlation of regional indicators: (**A**) the distribution of Italian regions by prevalence of SARS-CoV-2 positive cases, their trend, and the number of tests performed; and (**B**) the correlations between the prevalence of SARS-CoV-2 positive cases, their trend, and the number of tests performed; the results are reported as Spearman’s correlation coefficient from −1 (in red) to 1 (in green).

**Figure 2:**
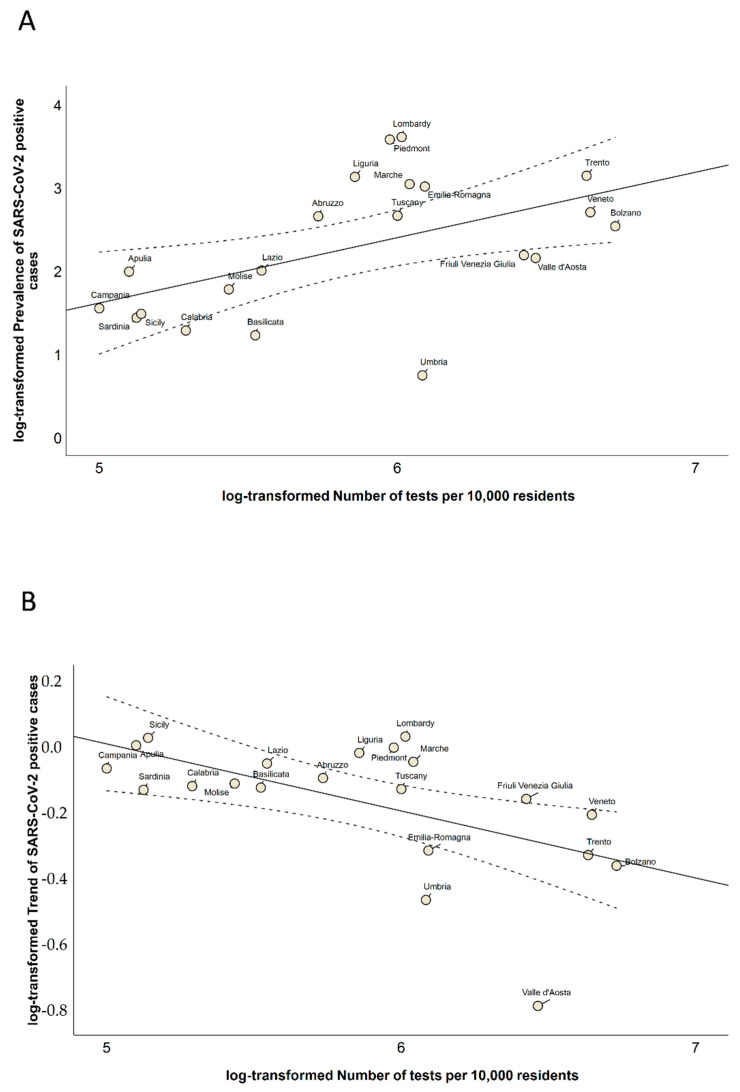
Scatter plots of the relationships of the number of tests performed with (**A**) the prevalence of SARS-CoV-2 positive cases and (**B**) their trend. Indicators are reported as log-transformed values.

In Figure 1A, we show how Italian regions were distributed in a three-dimensional scatter plot of these indicators. Although this plot showed no clear separation between the regions, we found that six and eight regions were above the national average for the prevalence and trend of SARS-CoV-2 positive cases, respectively. In contrast, 10 regions were below the national average for the number of tests performed per 10,000 residents. We also found a positive correlation between the number of tests performed and the prevalence of SARS-CoV-2 positive cases on 3 May 2020 (Spearman’s correlation coefficient = 0.527; *p* = 0.015). In contrast, we noted a significant negative correlation between the number of tests performed and the trend of SARS-CoV-2 positive cases in the week from 27 April to 3 May 2020 (Spearman’s correlation coefficient = −0.613; *p* = 0.003; Figure 1B). Specifically, we found a positive linear relationship between the log-transformed number of tests performed and the log-transformed prevalence of SARS-CoV-2 positive cases (β = 0.786; SE = 0.292; *p* = 0.015; Figure 2A). By contrast, the log-transformed trend of SARS-CoV-2 positive cases was negatively associated with the log-transformed number of tests performed (β = −0.201; SE = 0.067; *p* = 0.007; Figure 2B).

### 3.2. Regional Clusters

**Figure 3:**
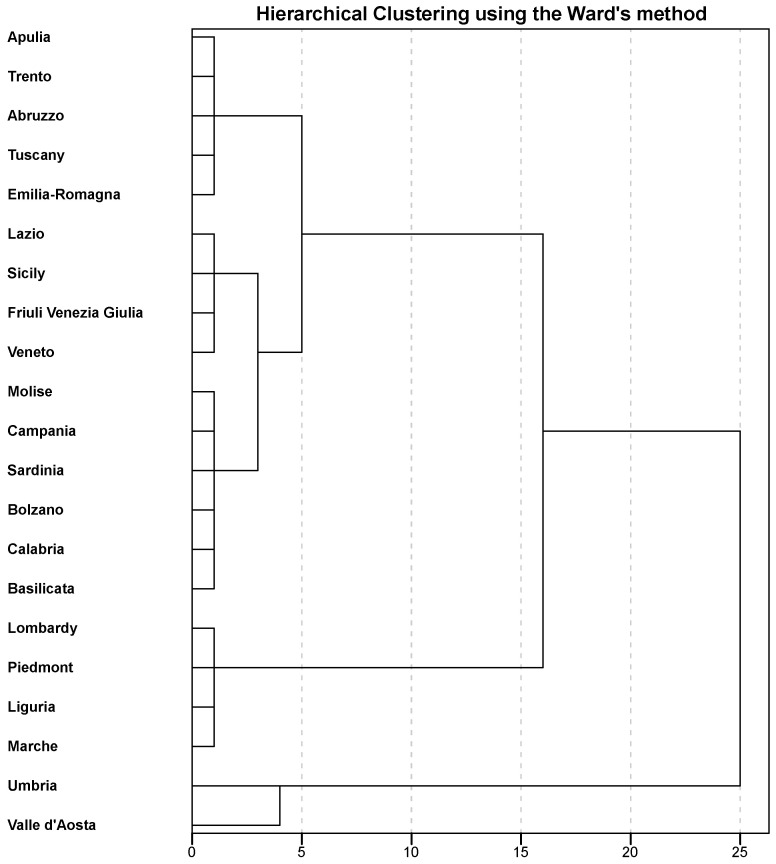
Dendrogram of the hierarchical clustering of regions based on Ward’s criterion.

We then applied hierarchical clustering, yielding to the dendrogram depicted in Figure 3. After the visual inspection of the hierarchical tree and based on silhouette values, we decided to partition Italian regions into three clusters. Indeed, the clustering solution with a number of three clusters was that with the highest average silhouette width (Table 2).

**Figure 4:**
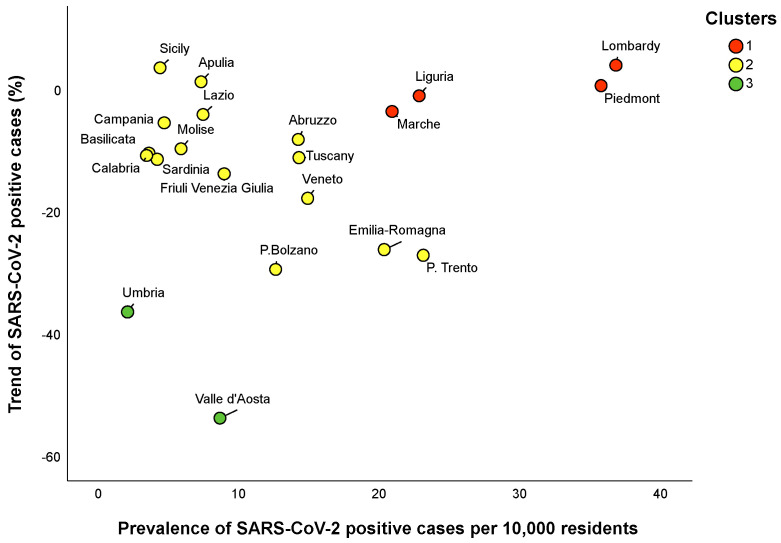
Scatter plot illustrating how the egional clusters were distributed on the prevalence of SARS-CoV-2 positive cases and their trend. Clustering solution was obtained by the hierarchical clustering and consolidated by the k-means algorithm.

According to this clustering solution, Lombardy, Piedmont, Liguria and Marche tended to be grouped into cluster 1, Umbria and Valle d’Aosta into cluster 3, and the remaining regions into cluster 2 (Figure 4). Differences between the clusters, in terms of the prevalence and trend of SARS-CoV-2 positive cases, are reported in Table 3. Specifically, the regions in cluster 1 exhibited the highest prevalence and trend of SARS-CoV-2 positive cases. In contrast, the regions in cluster 3 were those with the lowest prevalence and trend of SARS-CoV-2 positive cases. Regions in cluster 2 were those with an intermediate prevalence and trend of SARS-CoV-2 positive cases. We then consolidated this partition by applying a k-means algorithm with a predefined number of three clusters. However, all the regions maintained the same allocation of hierarchical clustering.

### 3.3. Provincial Clusters

**Figure 5:**
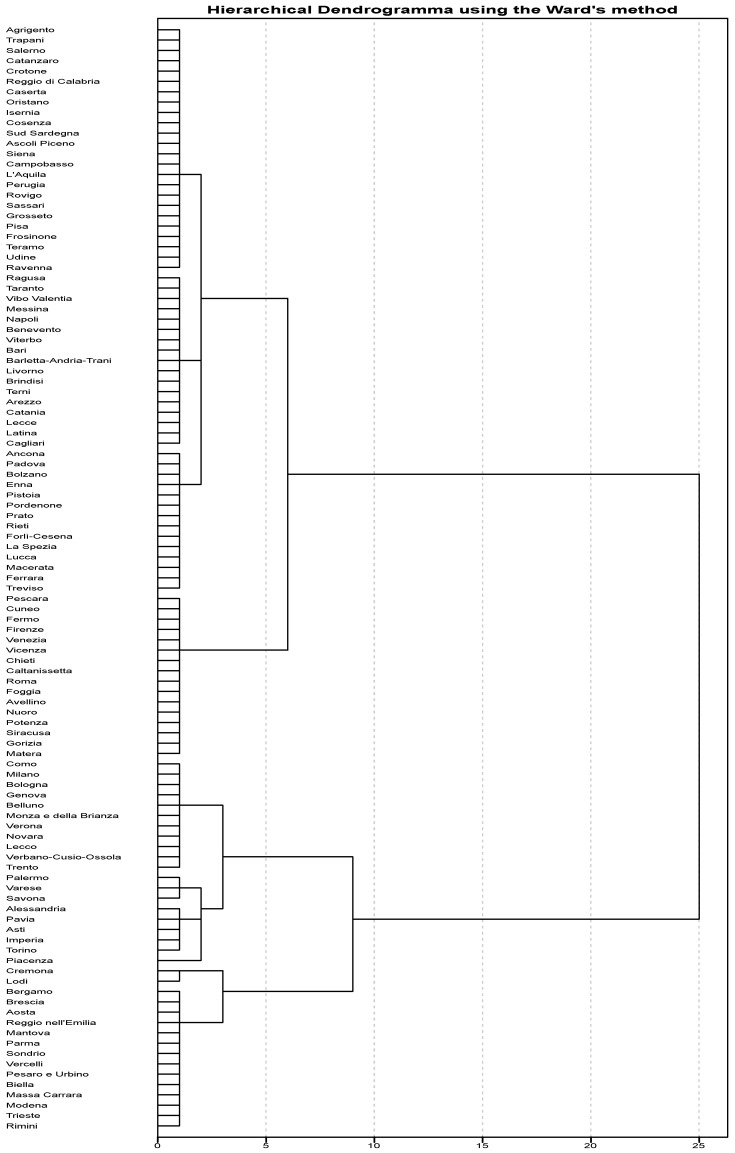
Dendrogram of the hierarchical clustering of the provinces based on Ward’s criterion.

We then applied a similar approach to identify different clusters among 107 Italian provinces, based on the prevalence and trend of total SARS-CoV-2 cases. The hierarchical clustering yielded to the dendrogram shown in Figure 5.

According to the highest silhouette value reported in Table 4, we obtained an optimal clustering solution by partitioning the provincial dataset into three clusters. The hierarchical clusters’ composition is summarized in Table 5.

**Figure 6:**
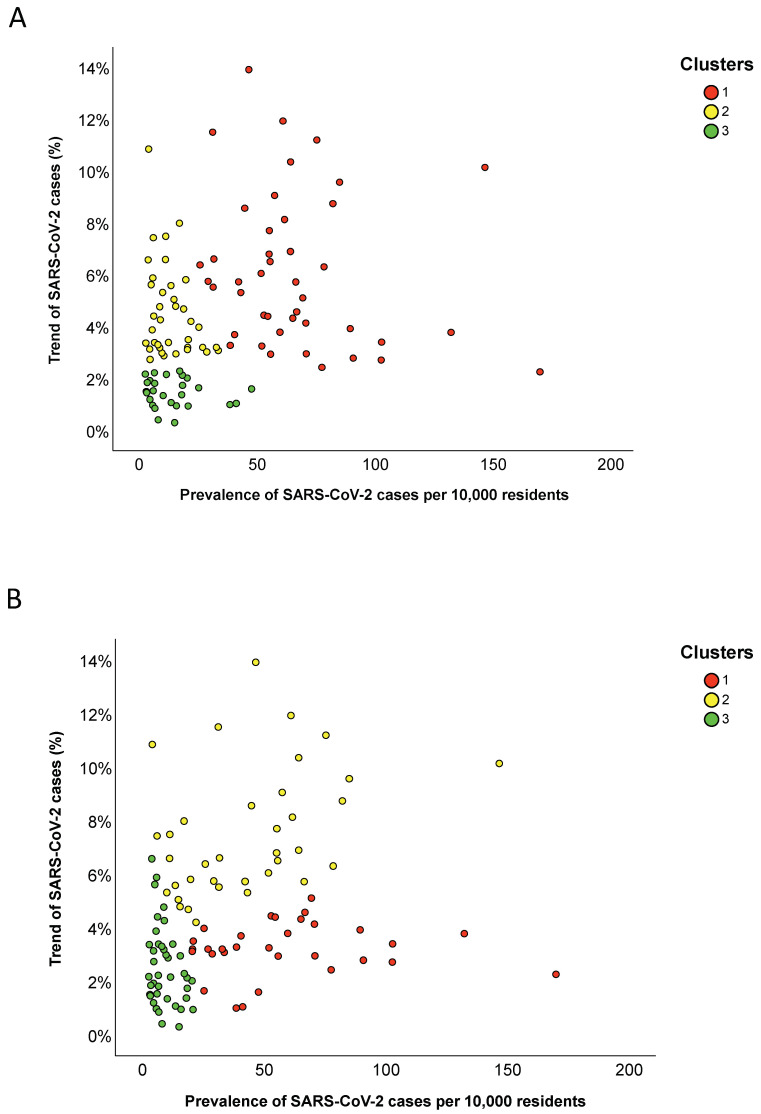
Scatter plot illustrating how provincial clusters were distributed on the prevalence of SARS-CoV-2 cases and their trend: (**A**) the clustering solution obtained by hierarchical clustering; and (**B**) the clustering solution obtained using the k-means algorithm.

Specifically, the provinces in cluster 1 exhibited the highest prevalence and trend of SARS-CoV-2 cases; the provinces in cluster 2 had a median prevalence of 11.2 cases per 10,000 residents (IQR = 14.0) and a median increment of 3.9% (IQR = 2.4); and the provinces in cluster 3 were those with the lowest trend and a median prevalence of 11.6 cases per 10,000 residents (IQR = 12.7) (Figure 6A and Table 6). We further consolidated the hierarchical clustering by applying the k-means algorithm with a predefined number of three clusters. This approach yielded to the alternative clustering solution summarized in Table 7. In particular, the provinces in cluster 1 exhibited the highest prevalence of SARS-CoV-2 cases and a median increment of 3.2% (IQR = 1.1); the provinces in cluster 2 had a median prevalence of 42.7 cases per 10,000 residents (IQR = 44.0) and the highest increment; the provinces in cluster 3 were those with the lowest prevalence and trend of SARS-CoV-2 cases (Figure 6B and Table 6).

## 4. Discussion

Data on the prevalence and trend of total cases could be useful to monitor the SARS-CoV-2 epidemic at the regional level [17]. To the best of our knowledge, our study was the first applying two clustering methods to categorize the Italian regions into different groups, based on the prevalence and trend of SARS-CoV-2 positive cases prior to the starting of phase two. Specifically, we first used hierarchical clustering [11] to identify the groups with a similar prevalence of SARS-CoV-2 positive cases on 3 May and trend from 27 April to 3 May 2020. According to this method, Lombardy and Piedmont—the most hit Italian regions [3]—were grouped with Umbria and Marche into cluster 1, which was characterized by a high prevalence and trend of SARS-CoV-2 positive cases. Looking at the data, the Umbria and Marche were characterized by the values of prevalence and trend that were hybrid between cluster 1 and 2. Most regions, instead, were classified into a more heterogeneous cluster, with an intermediate prevalence and trend of positive cases. Finally, Umbria and Valle d’Aosta—those regions that simultaneously exhibited a low prevalence and trend of active cases [3]—were grouped into cluster 3. We further applied the k-means algorithm, a partitioning clustering used for splitting the dataset into a predefined number of groups [11]. Our combined approach allowed to improve the robustness of our findings by using a mixed algorithm that produced a better clustering solution [11]. In this case, however, all the regions maintained the same allocation of hierarchical clustering.

Our approach did not intend to explore the causality of the observed differences between clusters, nor to account for the spatial distribution of SARS-CoV-2 cases within the Italian territory. Actually, we aimed to provide a simple tool to monitor the SARS-CoV-2 epidemic across Italian regions. Indeed, our findings offered a snapshot of the epidemic, which could be helpful to outline the hierarchy of needs at the regional level. Specifically, some Italian regions—Lombardy and Piedmont, surely, but also Liguria and Marche—required the highest level of attention. For all the other regions, there were hopeful signs that the epidemic was slowly going towards a resolution. Among these, interestingly, Umbria and Valle d’Aosta were those with the best scenario. There were several explanations as to why Italian regions exhibited different prevalences and trends of SARS-CoV-2 cases. First, the epidemic initially started in Lombardy and Veneto and only after spread to the other regions with different times and intensities [2,3]. Thus, temporal and spatial distributions of SARS-CoV-2 cases represented the first reason motivating the differences observed across Italian regions. Beyond this, it is possible to hypothesize other grounds to support our clustering solution, for instance, comparing the epidemic spread between Lombardy and Veneto regions. Indeed, the first outbreaks concomitantly but independently occurred in two small Italian areas within the Lombardy and Veneto regions [2]. However, the scenario observed in Lombardy on the 3 May 2020 was much more serious than that in Veneto. Interestingly, Veneto imposed a wider testing campaign than Lombardy (771 vs. 409 tests per 10,000 residents), which may have contributed to a more rapid resolution of the epidemic [18]. Moreover, it is worth mentioning that performing a lower number of tests might alter the statistics and sustain the epidemic spread through contacts of positive but undocumented SARS-CoV-2 patients [19,20]. Equally important, the variability in the level of compliance with national restrictions and recommendations between and within regions could have influenced their efficacy at the regional level, yielding to the observed differences between clusters [21]. Finally, the demographic structure of each region, as well as environmental factors, could represent more suggestive explanations underlying a part of the variation between regions.

Our study also intended to provide an optimal clustering solution at the provincial level, despite that the data released by Italian authorities were less accurate. Whilst it would have been proper to work on positive cases, we used available data on the prevalence of total cases on 3 May and their trend from 27 April to 3 May 2020. Most provinces, actually, were first included in a cluster with the highest prevalence and trend of total cases. The remaining provinces, instead, were partitioned into two clusters with a similar prevalence of SARS-CoV-2 cases (approximately 11 cases per 10,000 residents) but a different trend from 27 April to 3 May: provinces in cluster 2 exhibited a median increment of 3.9%, while those in cluster 3 had the lowest trend (i.e., 1.5%). An alternative clustering solution obtained by the k-means algorithm categorized Italian provinces in a cluster with the lowest prevalence and trend of SARS-CoV-2 cases and in two clusters with the highest prevalence or the highest trend, respectively. The aforementioned reasons supporting the differences between regions could be equally applied to the partition of Italian provinces.

Our study had several limitations that should be considered when interpreting our results. First, it did not intend to investigate the causality of the observed differences, but rather to provide a simple tool to classify the regions and provinces according to the prevalence and trend of SARS-CoV-2 cases prior to the start of phase two in Italy. In line, our approach did not take into account the temporal and spatial distributions of SARS-CoV-2 cases within the Italian territory. Moreover, it considered only a part of the available data and knowledge, and hence further analyses could include additional indicators, characteristics and epidemic parameters (e.g., demographic structure, environmental factors, adherence to national restrictions, mobility data) at the regional and provincial level that could improve our cluster solutions and motivate the observed partitions [22]. A main limitation was with regard to the accuracy of the data used in our analysis and the differences in ascertainment and reporting capabilities between and within regions. For instance, we did not consider the proportion of unreported cases [20,23,24,25], though previous studies suggested how undocumented SARS-CoV-2 cases could silently sustain the epidemic spread [23]. Indeed, the testing strategy adopted in Italy might have caused an underestimation of infectious individuals, especially those with mild or no symptoms [19]. For this reason, we adjusted the prevalence and trend indicators for the regional number of tests performed per 10,000 residents.

## 5. Conclusions

In view of these considerations, our study described a simple and immediate approach to monitor the SARS-CoV-2 epidemic at the regional and provincial level. Interestingly, we partitioned both Italian regions and provinces into several groups according to the prevalence and trend of SARS-CoV-2 cases. These findings, at present, could be important to support policymakers in monitoring and planning future strategies against the epidemic. Indeed, they offered a snapshot of the epidemic, which could be helpful to outline the hierarchy of needs at the subnational level. However, the integration of our approach with further indicators and characteristics (i.e., the spatial and temporal distribution of SARS-CoV-2 cases, compliance with national control measures, demographic structure, environmental factors, and mobility data) could improve our findings, also allowing the application to different contexts and with additional aims. To do that, further studies working on additional and more accurate data at the regional and provincial level should be encouraged.

## Figures and Tables

**Table 1:** Characteristics of the Italian regions.

Regions	Residents	Prevalence of Positive Cases (Per 10,000 Residents) ^a^	Trend of Positive Cases (%) ^b^	Number of Tests (Per 10,000 Residents) ^a^
Abruzzo	1,315,196	14.2	−8.00%	309.5
Apulia	4,048,242	7.3	1.50%	164.1
Basilicata	567,118	3.4	−10.60%	250.6
Bolzano ^c^	527,750	12.6	−29.30%	838.3
Calabria	1,956,687	3.6	−10.20%	198.5
Campania	5,826,860	4.7	−5.30%	148.5
Emilia-Romagna	4,452,629	20.3	−26.00%	442.6
Friuli Venezia Giulia	1,215,538	8.9	−13.60%	616.9
Lazio	5,896,693	7.4	−3.90%	255.9
Liguria	1,556,981	22.8	−0.80%	350
Lombardy	10,036,258	36.8	4.20%	409.4
Marche	1,531,753	20.9	−3.40%	420.5
Molise	308,493	5.9	−9.50%	229.3
Piedmont	4,375,865	35.7	0.80%	393.5
Sardinia	1,648,176	4.2	−11.20%	168.3
Sicily	5,026,989	4.4	3.80%	170.9
Trento ^c^	539,898	23.1	−27.00%	761.2
Tuscany	3,736,968	14.3	−11.00%	403.8
Umbria	884,640	2.1	−36.20%	438.9
Valle d’Aosta	126,202	8.6	−53.60%	641.8
Veneto	4,905,037	14.9	−17.60%	771.0

^a^ Data are referred to 3 May 2020. ^b^ Weekly trend from 27 April to 3 May 2020. ^c^ Autonomous provinces.

**Table 2:** Average silhouette width for the hierarchical clustering of regions.

Number of Clusters	Average Silhouette Width (Standard Deviation)
2	0.566 (0.166)
3	0.632 (0.099)
4	0.493 (0.197)
5	0.530 (0.248)

**Table 3:** Comparisons of the prevalence of SARS-CoV-2 positive cases, their trend and the number of tests between regional clusters.

Clusters	Prevalence of Positive Cases Per 10,000 Residents ^a^	Trend of Positive Cases ^b^	Number of Tests Per 10,000 Residents ^a^
Cluster 1	29.3 (15.2)	0% (6.1)	401.5 (56.9)
Cluster 2	7.4 (9.9)	−10.6% (12.3)	255.9 (446.0)
Cluster 3	5.4 (6.5)	−44.9% (17.4)	540.4 (202.9)
*p*-Value	0.011	0.007	0.356

Results are reported as the median (interquartile range), with *p*-values based on the Kruskal–Wallis test. ^a^ Data are referred to 3 May 2020 ^b^ Weekly trend from 27 April to 3 May 2020.

**Table 4:** Average silhouette width for the hierarchical clustering of provinces.

Number of Clusters	Average Silhouette Width (Standard Deviation)
2	0.399 (0.169)
3	0.402 (0.229)
4	0.368 (0.214)
5	0.377 (0.225)

**Table 5:** Hierarchical clusters’ composition of provinces.

Clusters	Provinces
Cluster 1	Alessandria; Aosta; Asti; Belluno; Bergamo; Biella; Bologna; Brescia; Como; Cremona; Cuneo; Fermo; Firenze; Forlì-Cesena; Genova; Imperia; La Spezia; Lecco; Lodi; Mantova; Massa Carrara; Milano; Modena; Monza e della Brianza; Novara; Parma; Pavia; Pesaro e Urbino; Pescara; Piacenza; Reggio nell’Emilia; Rimini; Savona; Sondrio; Torino; Trento; Trieste; Varese; Venezia; Verbano-Cusio-Ossola; Vercelli; Verona; Vicenza
Cluster 2	Arezzo; Avellino; Bari; Barletta-Andria-Trani; Benevento; Brindisi; Cagliari; Caltanissetta; Catania; Chieti; Enna; Ferrara; Foggia; Gorizia; Latina; Lecce; Livorno; Lucca; Macerata; Matera; Messina; Napoli; Nuoro; Palermo; Pistoia; Pordenone; Potenza; Prato; Ragusa; Rieti; Roma; Siracusa; Taranto; Terni; Treviso; Vibo Valentia; Viterbo
Cluster 3	Agrigento; Ancona; Ascoli Piceno; Bolzano; Campobasso; Caserta; Catanzaro; Cosenza; Crotone; Frosinone; Grosseto; Isernia; L’Aquila; Oristano; Padova; Perugia; Pisa; Ravenna; Reggio di Calabria; Rovigo; Salerno; Sassari; Siena; Sud Sardegna; Teramo; Trapani; Udine

**Table 6:** Comparisons of the prevalence of SARS-CoV-2 cases and their trend between clusters of provinces.

Clusters	Prevalence of Total Cases Per 10,000 Residents	Trend of Total Cases (%)
**Hierarchical Clustering**		
Cluster 1	61.0 (31.0)	5.7% (4.4)
Cluster 2	11.2 (14.0)	3.9% (2.4)
Cluster 3	11.6 (12.7)	1.5% (0.9)
*p*-Value	<0.001	<0.001
**K-means Clustering**		
Cluster 1	53.0 (38.1)	3.2% (1.1)
Cluster 2	42.7 (44.0)	6.7% (3.3)
Cluster 3	7.4 (8.5)	2.2% (2.0)
*p*-Value	<0.001	<0.001

Results are reported as the median (interquartile range), with *p*-values based on the Kruskal–Wallis test. Data are referred to 3 May 2020. Weekly trend from 27 April to 3 May 2020.

**Table 7:** K-means clusters’ composition of provinces.

Clusters	Provinces
Cluster 1	Ancona; Aosta; Bergamo; Biella; Bolzano; Brescia; Cremona; Enna; Ferrara; Forlì-Cesena; La Spezia; Lecco; Lodi; Lucca; Macerata; Mantova; Massa Carrara; Modena; Padova; Parma; Pesaro e Urbino; Pordenone; Prato; Ravenna; Reggio nell’Emilia; Rieti; Rimini; Sondrio; Treviso; Trieste; Vercelli
Cluster 2	Alessandria; Arezzo; Asti; Avellino; Belluno; Bologna; Brindisi; Caltanissetta; Chieti; Como; Cuneo; Fermo; Firenze; Foggia; Genova; Gorizia; Imperia; Matera; Milano; Monza e della Brianza; Novara; Palermo; Pavia; Pescara; Piacenza; Pistoia; Roma; Savona; Terni; Torino; Trento; Varese; Venezia; Verbano-Cusio-Ossola; Verona; Vicenza
Cluster 3	Agrigento; Ascoli Piceno; Bari; Barletta-Andria-Trani; Benevento; Cagliari; Campobasso; Caserta; Catania; Catanzaro; Cosenza; Crotone; Frosinone; Grosseto; Isernia; L’Aquila; Latina; Lecce; Livorno; Messina; Napoli; Nuoro; Oristano; Perugia; Pisa; Potenza; Ragusa; Reggio di Calabria; Rovigo; Salerno; Sassari; Siena; Siracusa; Sud Sardegna; Taranto; Teramo; Trapani; Udine; Vibo Valentia; Viterbo
